# RNA-Seq analysis of *Clerodendrum inerme* (L.) roots in response to salt stress

**DOI:** 10.1186/s12864-019-6098-y

**Published:** 2019-10-10

**Authors:** Yuping Xiong, Haifeng Yan, Hanzhi Liang, Yueya Zhang, Beiyi Guo, Meiyun Niu, Shuguang Jian, Hai Ren, Xinhua Zhang, Yuan Li, Songjun Zeng, Kunlin Wu, Feng Zheng, Jaime A. Teixeira da Silva, Guohua Ma

**Affiliations:** 1 Guangdong Provincial Key Laboratory of Applied Botany, South China Botanical Garden, the Chinese Academy of Sciences, Guangzhou, 510650 China; 2 University of Chinese Academy of Sciences, Beijing, 100039 China; 3 Cash Crop Institute of Guangxi Academy of Agricultural Sciences, Nanning, 30007 China; 4 Miki-cho Post Office, P.O. Box 7, Ikenobe 3011-2, Miki-cho, Kagawa-ken 761-0799 Japan

**Keywords:** Halophyte, *Clerodendrum inerme* (L.) Gaertn, Salt stress, Transcriptome

## Abstract

**Background:**

*Clerodendrum inerme* (L.) Gaertn, a halophyte, usually grows on coastal beaches as an important mangrove plant. The salt-tolerant mechanisms and related genes of this species that respond to short-term salinity stress are unknown for us. The de novo transcriptome of *C. inerme* roots was analyzed using next-generation sequencing technology to identify genes involved in salt tolerance and to better understand the response mechanisms of *C. inerme* to salt stress.

**Results:**

Illumina RNA-sequencing was performed on root samples treated with 400 mM NaCl for 0 h, 6 h, 24 h, and 72 h to investigate changes in *C. inerme* in response to salt stress. The de novo assembly identified 98,968 unigenes. Among these unigenes, 46,085 unigenes were annotated in the NCBI non-redundant protein sequences (NR) database, 34,756 sequences in the Swiss-Prot database and 43,113 unigenes in the evolutionary genealogy of genes: Non-supervised Orthologous Groups (eggNOG) database. 52 Gene Ontology (GO) terms and 31 Kyoto Encyclopedia of Genes and Genomes (KEGG) pathways were matched to those unigenes. Most differentially expressed genes (DEGs) related to the GO terms “single-organism process”, “membrane” and “catalytic activity” were significantly enriched while numerous DEGs related to the plant hormone signal transduction pathway were also significantly enriched. The detection of relative expression levels of 9 candidate DEGs by qRT-PCR were basically consistent with fold changes in RNA sequencing analysis, demonstrating that transcriptome data can accurately reflect the response of *C. inerme* roots to salt stress.

**Conclusions:**

This work revealed that the response of *C. inerme* roots to saline condition included significant alteration in response of the genes related to plant hormone signaling. Besides, our findings provide numerous salt-tolerant genes for further research to improve the salt tolerance of functional plants and will enhance research on salt-tolerant mechanisms of halophytes.

## Background

Soil salinization is a serious threat to the environment and agricultural productivity worldwide. Research into salt tolerance and its underlying mechanisms has been carried out in many plant species, such as rice [1, 2], melon [3], maize [4], tobacco [5] and tomato [6]. Investigations of plant salt tolerance are crucial for the sustainable development of saline agriculture. To achieve this, salt-tolerant species are essential for salt stress research [7, 8]. Halophytes, which account for about 1% of the world’s total flora, can survive in highly saline environments and possess salt-responsive genes and proteins to encounter the adverse effects of salinity. Halophytes are further divided into hydro-halophytes and xero-halophytes. Hydro-halophytes grow in aquatic conditions or wet soil, such as mangroves and salt-marsh species along coastlines whereas xero-halophytes grow in habitats where the soil is always saline and dries out periodically causing water to become unavailable to the plant, such as succulent species in desert areas [9, 10]. Having natural salt tolerance, halophytic species represent ideal material to explore complex physiological and molecular mechanisms of salt tolerance, such as *Thellungiella halophila* [11, 12], *Halogeton glomeratus* [13] and *Suaeda glauca* [14].

The mechanisms of salt tolerance in halophytes have been gradually revealed over the past few years. The salt tolerance of a halophyte is determined by effective coordination between various physiological processes, metabolic pathways and gene networks under salt stress, including activation of antioxidant enzymes, induction and modulation of plant hormones, biosynthesis of compatible solutes and osmoprotectants, selective accumulation, exclusion of ions, and other mechanisms [15–18]. Furthermore, numerous genes that were isolated from halophytes have also been exploited by various modern biotechnological techniques to identify the genes that function in salt tolerance, including genes encoding for transcription factors [19, 20], the Na^+^/H^+^ antiporter gene [21, 22] and genes encoding antioxidant enzymes [23, 24]. The existence of these genes enhances the tolerance of plants to salt and provides more choices for the improvement of salt tolerance in crops. However, considering the variety of halophyte species, our understanding of the mechanism underlying salt tolerance in halophyte is limited. Novel processes or genes may exist in different halophyte species.

*Clerodendrum inerme* (L.) Gaertn, a synonym of *Volkameria inermis* L. (http://www.theplantlist.org/; last accessed May 28, 2019), which is distributed in the South of China, India and Southeast Asia to Northern Oceania, usually grows on coastal beaches as a mangrove plant and be used for coastal afforestation. In fact, *C. inerme* also displays tremendous potential for ecological development. A few reports of *C. inerme* have indicated its value based on phytochemical composition [25–27] and medicinal properties [28–30]. *C. inerme* is used by coastal people as a poultice for skin disease and to treat wounds [31]. Not only it has been listed as a hydro-halophyte for use in saline agriculture in Pakistan [32], but it has also been recorded as a medicinal halophyte in the Chinese Germplasm Resources of Halophytes (http://www.grhc.sdnu.edu.cn/; last accessed May 28, 2019). Several mangrove species with exceptional salt tolerance have been reported, including *Rhizophora stylosa* [33], *Sonneratia alba* [34] and *Avicennia officinalis* [35]. Novel genes that involved in salt response of those species revealed in above reports. As a mangrove species with important ecological and scientific value, salt tolerance mechanism in *C. inerme* is unclear. The potential candidate genes have not been identified in this halophyte species.

In recent years, RNA-seq, as an important strategy in the investigation of the expression of a large number genes in a given tissue at a given time point, has been widely used to research the responses of plants to abiotic stress [36, 37], particularly salt stress [38, 39]. And de novo assembly makes it possible for a detailed genetic analysis to be performed on any organism [40] and has been applied to a number of halophytes to uncover their mechanisms of salt tolerance. This technology can reveal candidate genes and key pathways involved in salt tolerance by analyzing differentially expressed genes (DEGs) and RNA-seq functional annotation. Transcriptome analysis reveals that several levels of biochemical and molecular responses are associated with salt tolerance in *Suaeda maritima*, including the remodeling of cell walls and the modification of membrane lipids at the structural level and the accumulation of glycine betaine and the sequestration and exclusion of Na^+^ at the cellular level [41]. Genes related to membrane transport, osmoprotection, redox metabolism or protein synthesis were differentially expressed in *Beta vulgaris ssp. maritima* in response to salt stress [42]. In *Glehnia littoralis*, DEGs encoding transcription factors or related to plant hormone signaling, calcium signaling, and phospholipase signaling, were responsive to NaCl treatment [43]. RNA-seq technology assists researchers to establish a better understanding of molecular networks in plant in response to salt stress.

## Results

### Effect of salt stress on activity of antioxidant enzymes, H_2_O_2_ content and ion content

**Fig. 1:**
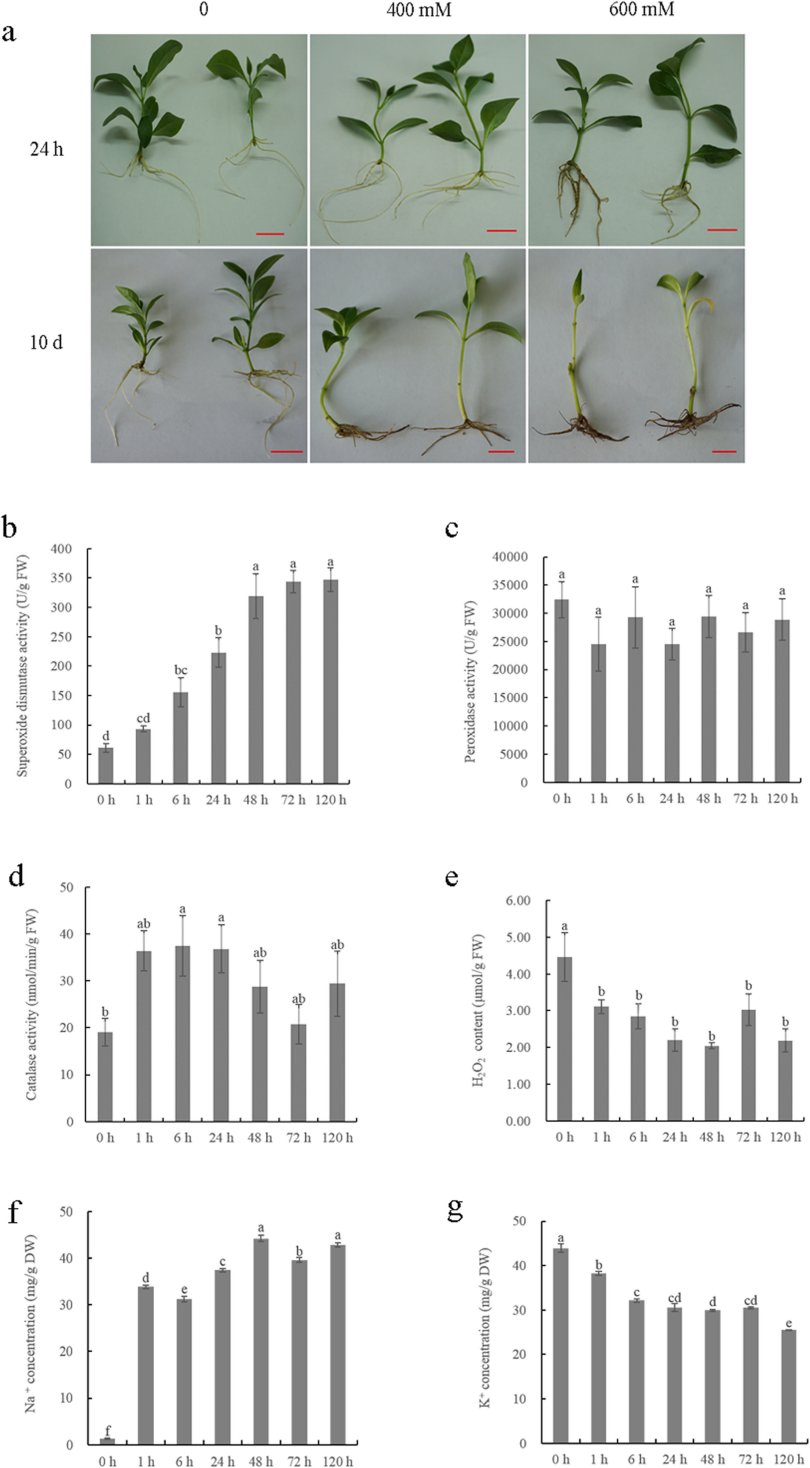
Effect of NaCl treatment on *Clerodendrum inerme* growth. (**a**) Growth phenotype of *C. inerme* under control and stress conditions. The red bars represent one centimeter. (**b**, **c**, **d**, **e**, **f** and **g**) Root antioxidant enzyme activity (**b**: SOD, **c**: POD, **d**: CAT), H_2_O_2_ content (**e**) and Na^+^ (**f**), K^+^ (**g**) content in *C. inerme* at 0 h, 1 h, 6 h, 24 h, 48 h, 72 h and 120 h after treatments. Different letters indicate statistically significant differences based on Duncan’s multiple range test (*P* < 0.05) for the designated treatments

Under 600 mM NaCl treatment, the roots of *C. inerme* turned brown after 24 h treatment and gradually grow wilt. While, under 400 mM NaCl treatment, no visible damage were observed on *C. inerme* roots after 24 h treatment. The roots grow lightly brown at 10 days (Fig. 1a). Thus, 400 mM NaCl was chosen for the further test. In the 400 mM NaCl treatment, root superoxide dismutase (SOD) and catalase (CAT) activity increased sharply and a sharp increase in SOD activity was observed from 6 h to 48 h, but CAT activity decreased after 24 h. Unlike SOD and CAT, root peroxidase (POD) activity changed slightly and showed no significant changes after NaCl treatment (Fig. 1b, c, d). In contrast, hydrogen peroxide (H_2_O_2_) content continued to decline after 1 h treatment (Fig. 1e). The Na^+^ content was significantly higher in NaCl-treated roots than in the control, and increased sharply at 6 h, 24 h treatment. After a peak at 48 h treatment, a decline of Na^+^ content was observed in *C. inerme* roots at 72 h treatment (Fig. 1f). By contrast, the K^+^ content showed significant decline under NaCl treatment. The downtrend became slow after 6 h treatment (Fig. 1g). The wild fluctuations of antioxidant enzymes activity and ion accumulation in *C. inerme* roots under NaCl stress suggest that a substantial change in salt responsive gene expression might occur at 6 h, 24 h and 72 h after 400 mM NaCl treatment. Therefore, these root samples (i.e., 0 h, 6 h, 24 h and 72 h) were subjected to RNA-seq analysis.

### De novo assembly and quality assessment of transcriptome

**Fig. 2:**
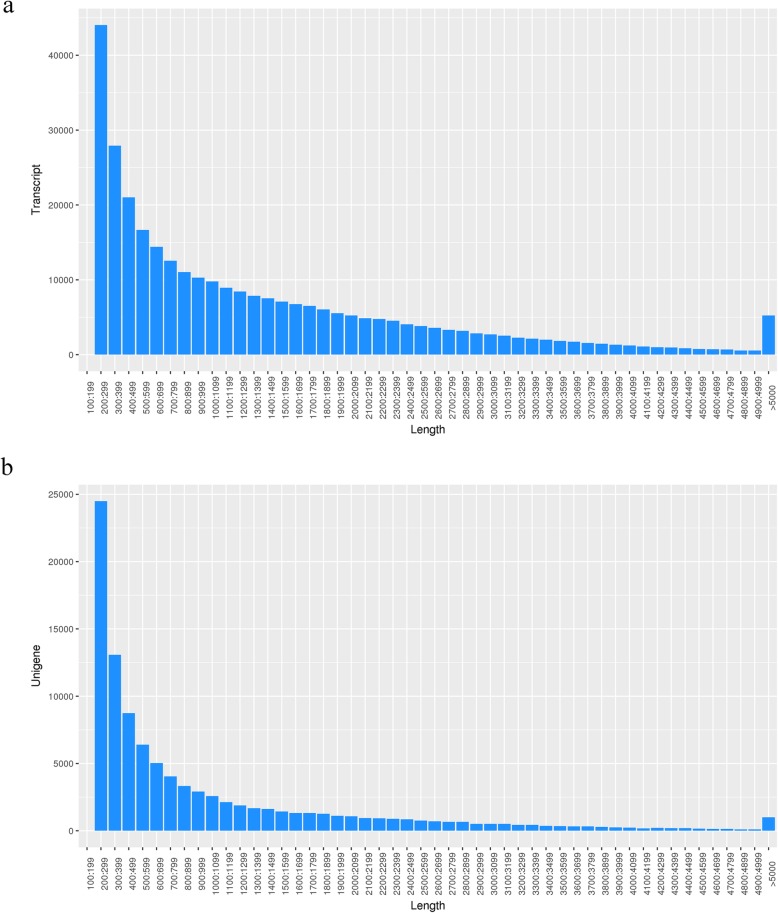
Random distribution of transcript (**a**) and unigene (**b**) length in *Clerodendrum inerme*. The x-axis indicates length of the transcript or unigene. The y-axis indicates the number of transcripts or unigenes

Twelve cDNA libraries prepared from three repeat RNA samples from the 0 h, 6 h, 24 h, and 72 h treatment were sequenced on the Illumina HiSeq platform and 692,669,572 raw reads were obtained. The sequenced raw data in this article was submitted to the SRA at the NCBI database with the following accession numbers: SRR8203779, SRR8203780, SRR8203777, SRR8203778, SRR8203783, SRR8203784, SRR8203781, SRR8203782, SRR8203785, SRR8203786, SRR8203775, and SRR8203776. After removing low-quality regions and adapters, we obtained a total of 683,948,928 clean reads with Q20 > 96.11% (Table 1). The quality of transcripts and unigene length distribution are shown in Fig. 2.

**Table 1:** Statistics of output sequencing in *Clerodendrum inerme*

Samples	Reads (No.)	Clean reads (No.)	Bases (bp)	Clean data (bp)	N (%)	Q20 (%)
0 h-1	60,802,270	60,008,714	9,120,340,500	8,912,712,572	0.00139	97.57
0 h-2	57,493,894	56,789,814	8,624,084,100	8,431,598,540	0.001364	97.69
0 h-3	56,866,282	55,934,266	8,529,942,300	8,313,133,758	0.002491	96.21
6 h-1	55,327,066	54,686,334	8,299,059,900	8,098,537,576	0.001451	97.79
6 h-2	55,683,662	55,033,118	8,352,549,300	8,155,979,860	0.001475	97.8
6 h-3	58,976,774	58,281,832	8,846,516,100	8,646,526,566	0.001437	97.73
24 h-1	60,813,298	60,098,036	9,121,994,700	8,903,035,660	0.001583	97.79
24 h-2	57,868,180	57,194,542	8,680,227,000	8,506,194,364	0.001434	97.72
24 h-3	58,931,324	58,021,340	8,839,698,600	8,634,386,222	0.002948	96.11
72 h-1	55,592,334	54,926,346	8,338,850,100	8,166,663,782	0.001337	97.57
72 h-2	57,207,480	56,526,470	8,581,122,000	8,408,351,478	0.001331	97.53
72 h-3	57,107,008	56,448,116	8,566,051,200	8,386,908,366	0.00143	97.63

Reads (No.) = total number of reads. Clean reads (No.) = number of high quality sequence reads. Bases (bp) = total number of bases. Clean data = number of high quality sequence bases. The Q20 percentage is the proportion of nucleotides with a quality value > 20. N (%) = the proportion of unknown nucleotides in clean reads

### Transcriptome annotation

**Fig. 3:**
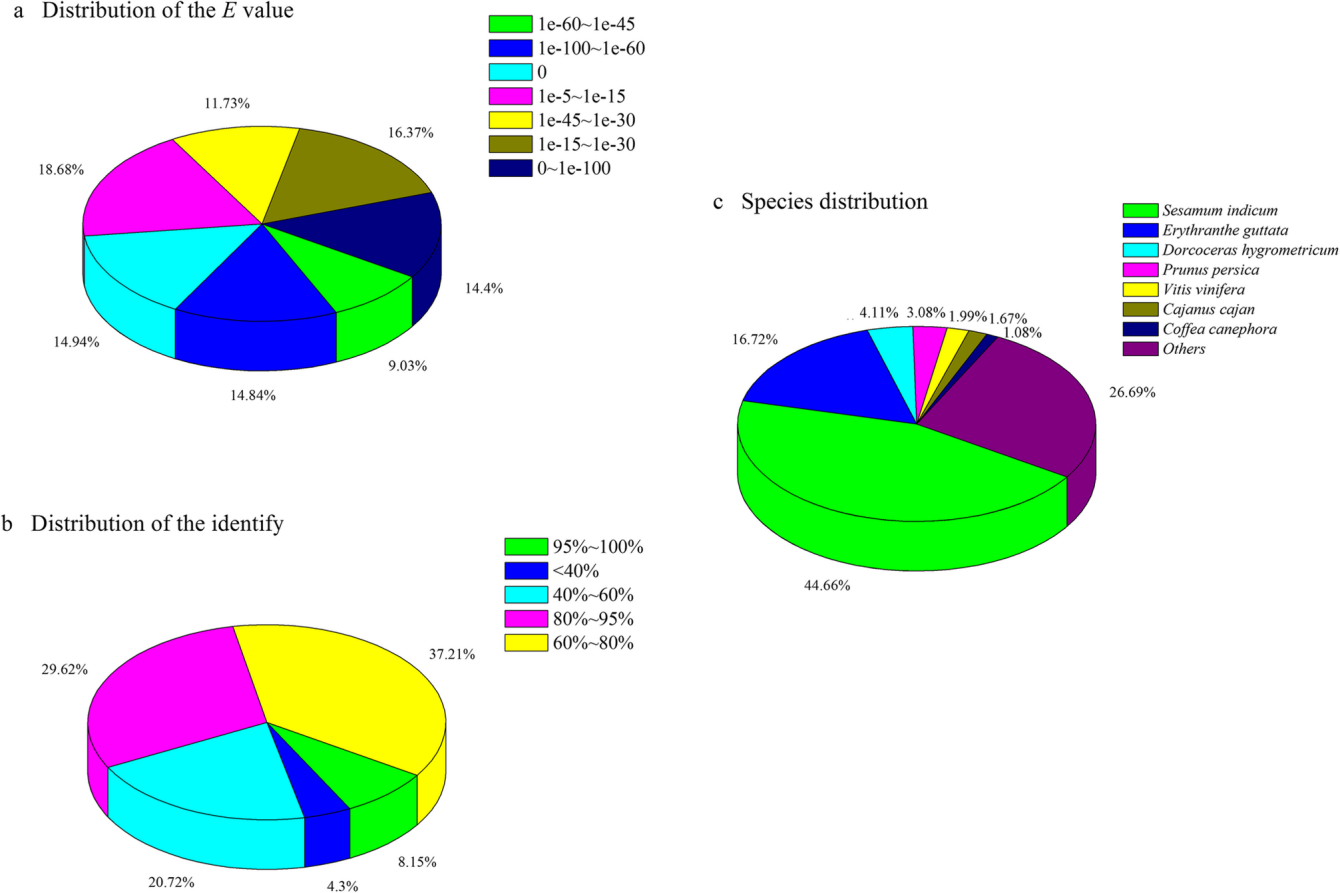
A homology search of unigenes against the NR database in *Clerodendrum inerme*. (**a**) *E*-value distribution of NR annotation result. (**b**) Distribution of the identification. (**c**) Species distribution of the top BLAST hits. Pie charts were generated by Origin 7.5

Using the best hits found by BLAST with *E* values <1e-5, an inferred putative function was assigned to the sequences. A total of 46,085 (46.57%) unigenes were matched to known genes in the NR database, and 34,756 (35.12%) sequences had best hits in the Swiss-Prot database (Table 2). As shown in Fig. 3, the *E*-value distribution of the top hits in the NR database showed that 38.28% of the sequences were mapped to known genes in plants with best hits (*E* value <1e-45) (Fig. 3a), and approximately 37.76% of the unigenes showed > 80% similarity with deposited sequences (Fig. 3b). Approximately 73.31% of the annotated unigenes were assigned with a best score to sequences from the top seven species (Fig. 3c): *Sesamum indicum* (44.66%), *Erythranthe guttata* (16.72%), *Dorcoceras hygrometricum* (4.11%), *Prunus persica* (3.08%), *Vitis vinifera* (1.99%), *Cajanus cajan* (1.67%) and *Coffea canephora* (1.08%).

**Table 2:** The results of annotation in *Clerodendrum inerme*

Annotation databases	Isoform numbers	Percentage (%)
NR	46,085	46.57
GO	22,473	22.71
KEGG	5969	6.03
eggNOG	43,113	43.56
Swissprot	34,756	35.12
In all databases	4396	4.4

**Fig. 4:**
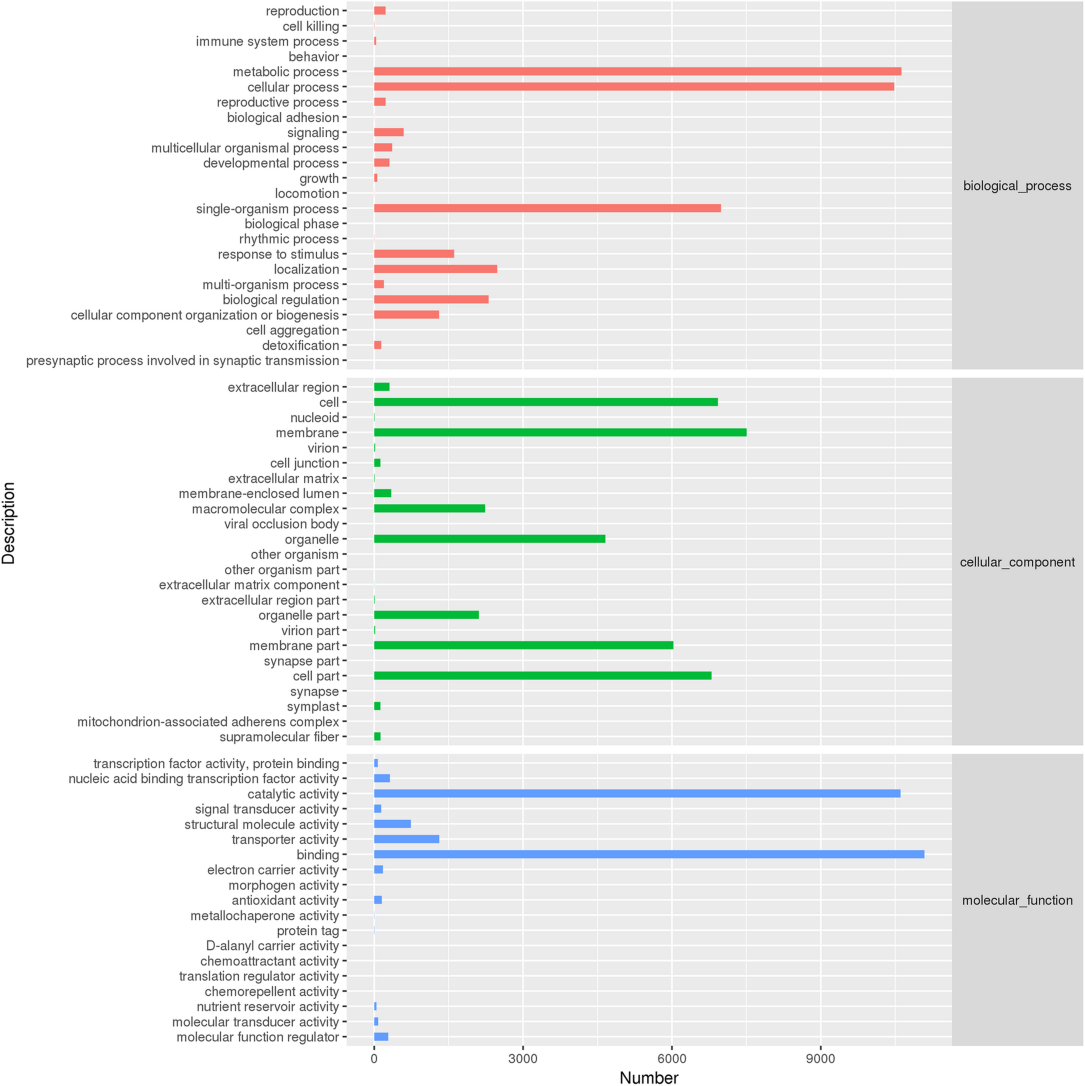
GO classification of all unigenes in *Clerodendrum inerme.* Graphs were generated by R software

A total of 22,473 unigenes were assigned to GO terms that described biological processes, molecular functions, and cellular components (Fig. 4). Most annotated unigenes in biological processes were involved in “metabolic process”, “cellular process”, ‘single-organism process”, and “localization and biological regulation”. In the category of molecular functions, the highest portion of annotated unigenes were enriched in terms of “binding”, “catalytic activity” and “transporter activity”. In the cellular component, more annotated unigenes were categorized in the “membrane”, “cell” and “cell part”.

**Fig. 5:**
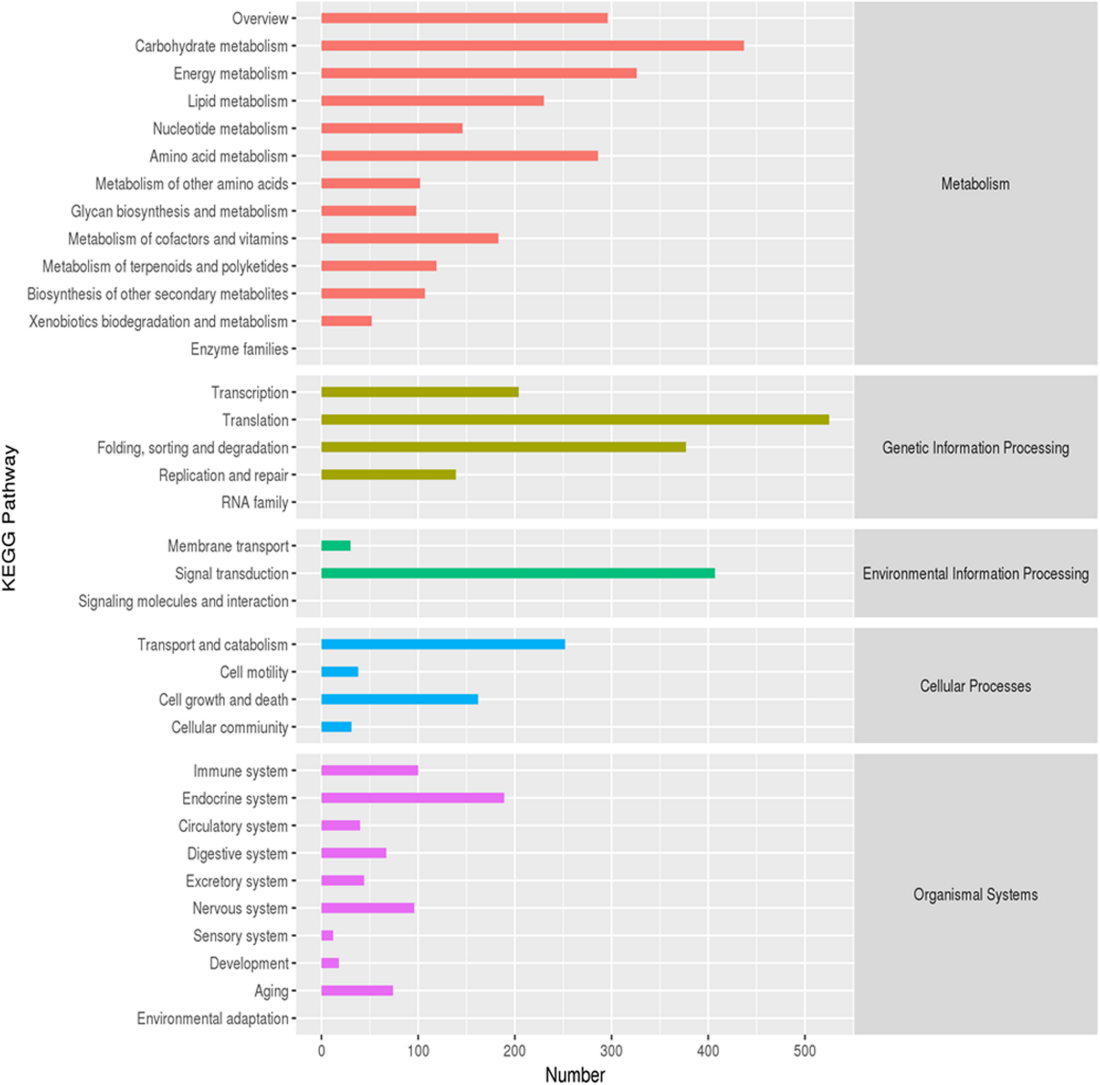
KEGG pathway analysis of all unigenes in *Clerodendrum inerme.* Graphs were generated by R software

We obtained a KEGG pathway annotation for 5969 unigenes, and 31 pathways were assorted based on five branches, including metabolism, genetic information processing, environmental information processing, cellular processes and organismal systems (Fig. 5). On the basis of KEGG analysis, most unigenes were annotated into sub-branches of “carbohydrate metabolism”, “translation” and “signal transduction”.

**Fig. 6:**
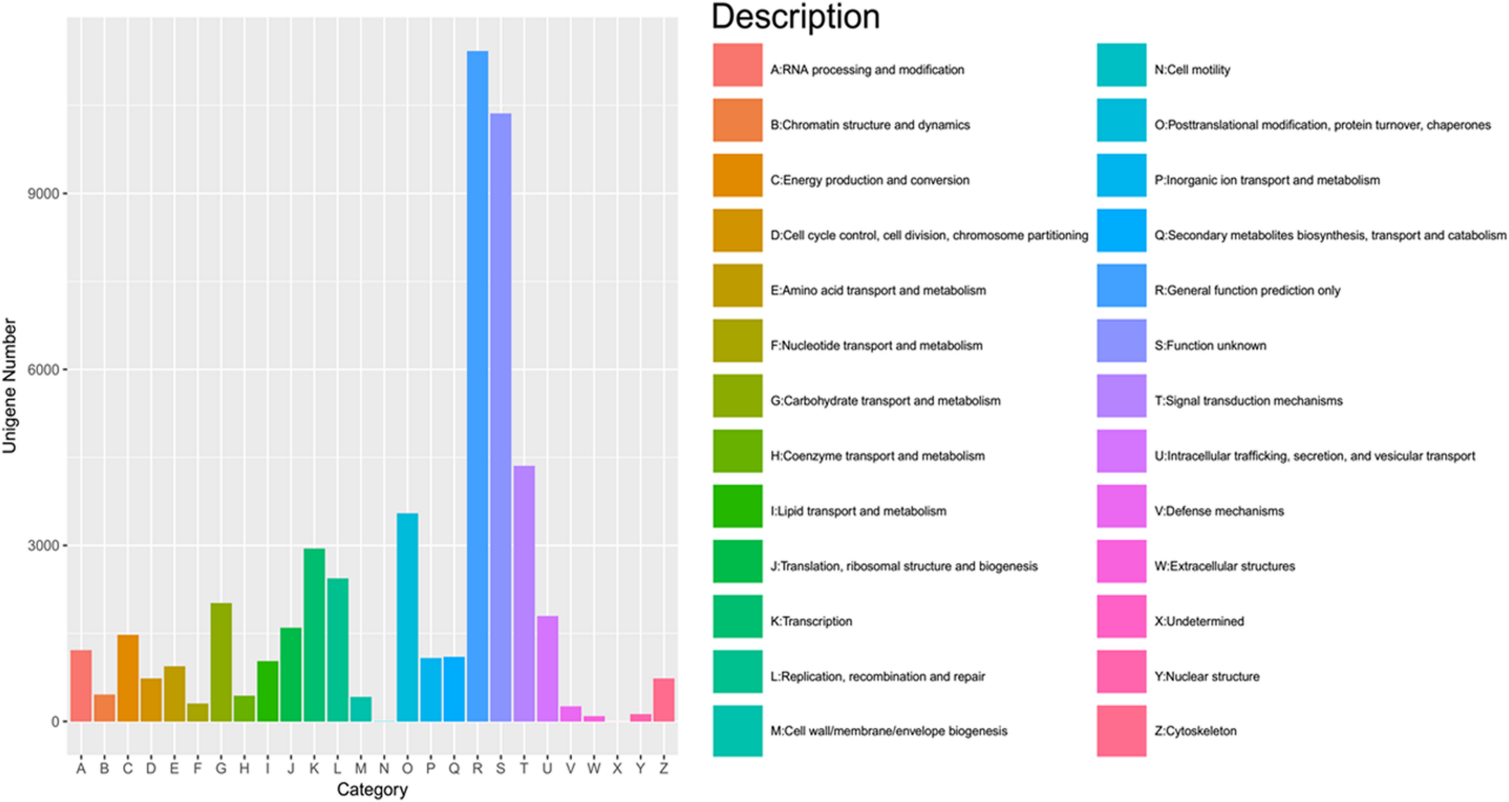
eggNOG function classification of all unigenes in *Clerodendrum inerme*. Graphs were generated by R software

Unigenes were also aligned to the eggNOG database to predict and classify possible functions. A total of 43,113 unigenes were distributed into 25 categories (Fig. 6). Among them, the NOG category “general function prediction only” represented the largest group, followed by “function unknown”, “signal transduction mechanisms”, “posttranslational modification, protein turnover, chaperones”, “transcription”, “replication, recombination and repair”, and “carbohydrate transport and metabolism”. The smallest group was “cell motility”.

### Gene expression and analysis of differentially expressed genes

The analysis of gene expression density (Additional file 6 : Fig. S1) showed that most transcripts were activated by salt stress, and the log10 (FPKM) ranged from 0 to 1, as assessed by boxplot analysis (Additional file 7 : Fig. S2). The operational stability and reliability of this experiment were illustrated with a high Pearson’s correlation coefficient (*R*^2^ > 0.9) among the three biological replicates at each time point (Additional file 8 : Fig. S3). These analyses suggested that the gene expression levels in this experiment were reliable for the next step of the analysis.

**Fig. 7:**
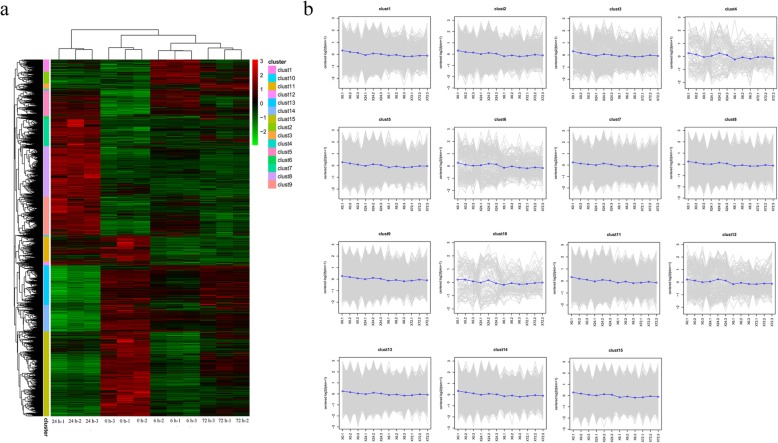
*Clerodendrum inerme* hierarchical cluster analysis of DEGs in different treatments. (**a**) Heat map of the expression profiles of DEGs. (**b**) The expression analysis of DEGs in 15 clusters. The x-axis represents the time point after each treatment with three replicates, while the y-axis represents the value of the expression level (log2(FPKM+ 1))

**Fig. 8:**
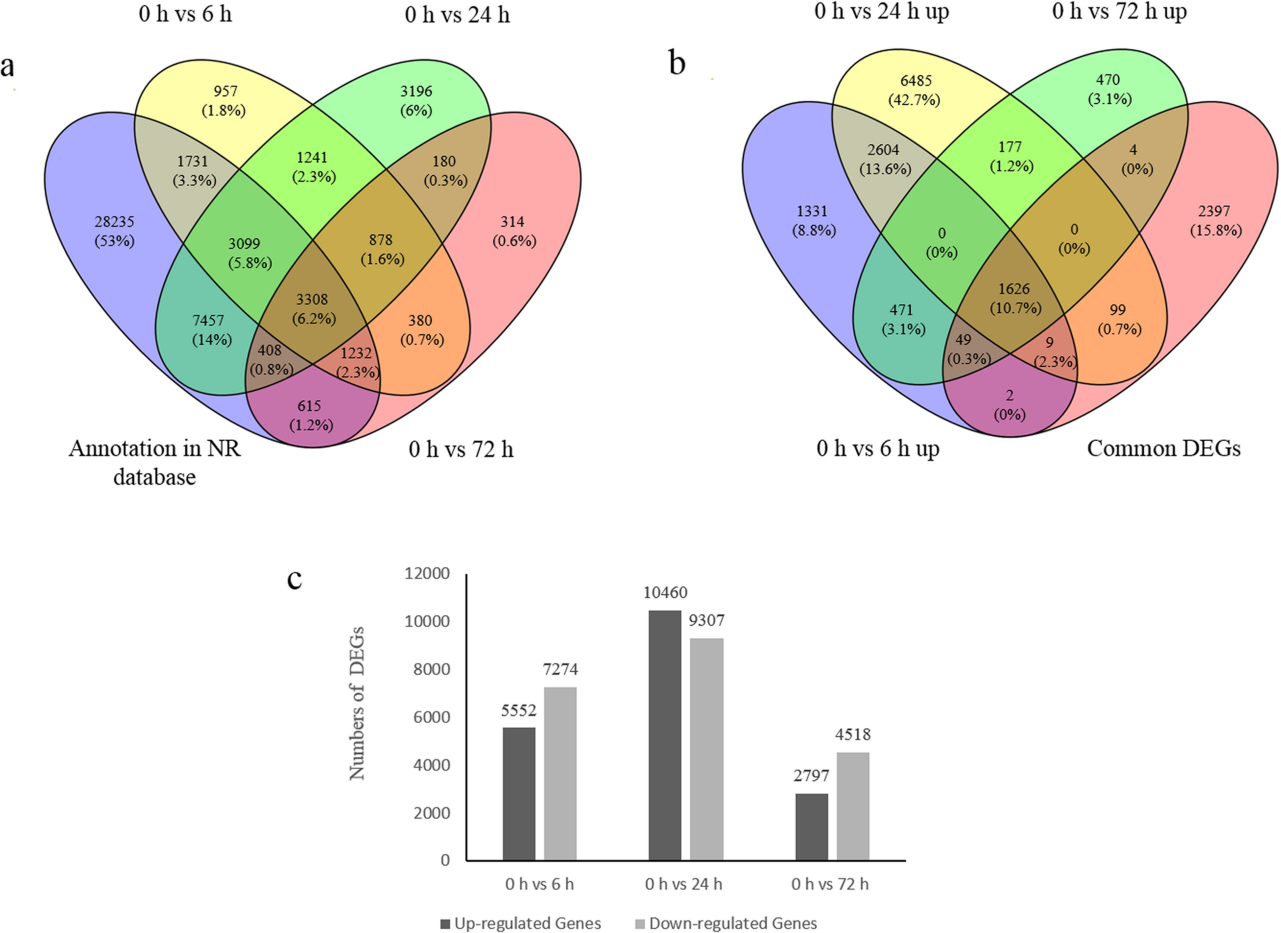
Venn diagram analysis of DEGs and the number of DEGs at 6 h, 24 h, and 72 h treatment compared with 0 h in *Clerodendrum inerme*. Venn diagrams were generated by Venn 2.1.0. (**a**) Venn diagram analysis of DEGs that were annotated in the NR database. (**b**) Venn diagram analysis of the up-regulated unigenes, and the DEGs that were up-regulated at at least two stages. (**c**) The number of up-regulated and down-regulated DEGs in 6 h, 24 h, and 72 h treatment compared with 0 h

Hierarchical clustering was used to analyze the expression patterns of DEGs under different experimental conditions. As shown in Fig. 7, the DEGs were divided into 15 main clusters. The highest number of up-regulated genes was observed at 24 h (10,460) and fewest at 72 h (2,797). The percentage of annotated DEGs in the NR database in the 6 h, 24 h, and 72 h libraries was 73.06, 72.20 and 76.05%, respectively. A total of 4186 common DEGs were obtained from 6 h, 24 h, and 72 h libraries (Fig. 8a), including 1626 up-regulated genes in all stages (Fig. 8b). There were many more DEGs at 24 h (19,767) than at 6 h (12,826) and 72 h (7,315) (Fig. 8c).

### GO term and KEGG pathway enrichment analysis of DEGs

Although the plantlets were treated with NaCl for different periods of time, the significant enrichment GO terms in those stages was very similar (Additional file 1 : Table S1). In biological processes, the dominant term was “single-organism process”. In the cellular component, the dominant term was “membrane”. Among the molecular functions, the dominant term was “catalytic activity”. The common DEGs were significantly enriched more in “single-organism metabolic process” and “oxidation-reduction process” of biological processes, “membrane” and “membrane part” of the cellular component, and “catalytic activity” and “cation binding” of molecular functions. Otherwise, the number and categories of extremely enriched GO terms differed between stages. At 6 h, 53 enriched GO terms were identified, in which “oxidation-reduction process”, “intrinsic component of membrane” and “oxidoreductase activity” term were highly enriched. At 24 h, 25 enriched GO terms were identified, in which “recognition of pollen”, “integral component of membrane” and “catalytic activity” term were highly enriched. The most number of enriched GO terms was observed at 72 h, with 79 GO terms related to “oxidation-reduction process”, “photosystem” and “tetrapyrrole binding” being highly enriched.

**Fig. 9:**
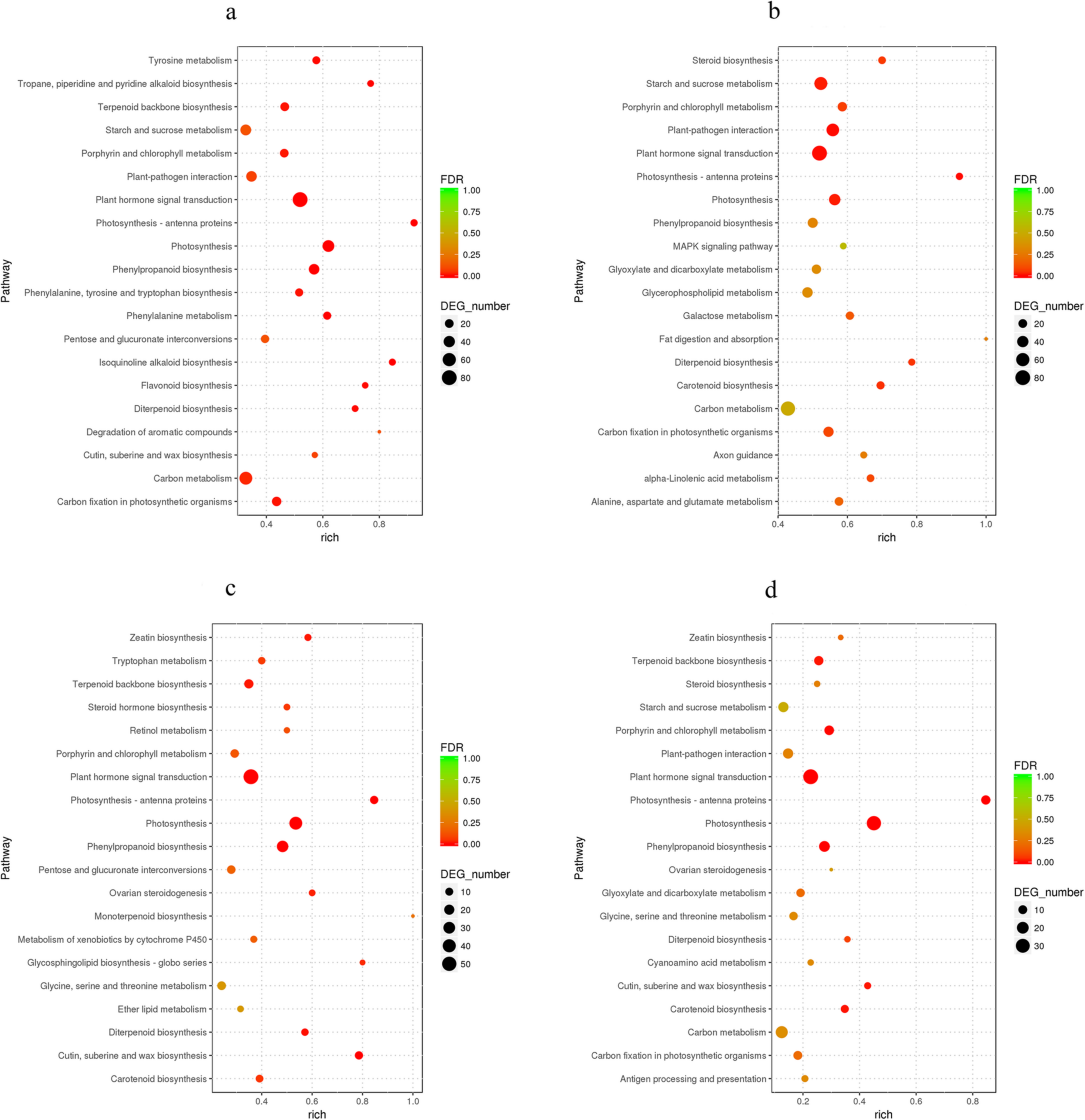
KEGG pathway enrichment analysis of DEGs in *Clerodendrum inerme*. (**a**) 0 h vs 6 h; (**b**) 0 h vs 24 h; (**c**) 0 h vs 72 h. (**d**) KEGG pathway enrichment of common DEGs at 6 h, 24 h, and 72 h. Graphs were generated by R software

Compared with 0 h, the DEGs of 6 h, 24 h, and 72 h libraries were assigned to 242, 261, and 209 sub-branches of the KEGG pathway, respectively. The significantly enriched pathways in NaCl-treated samples at 6 h, 24 h, and 72 h were shown in Fig. 9 and Additional file 2 : Table S2. Unlike the enriched GO terms, the number of enriched pathways peaked at 6 h with 40 pathways, followed by 72 h with 23 pathways, and 24 h with 22 pathways. The enriched pathways with more genes than other pathways were similar in 6 h, 24 h, and 72 h libraries, including “plant hormone signal transduction”, “carbon metabolism”, “photosynthesis”, “phenylpropanoid biosynthesis”, “starch and sucrose metabolism”, and “plant-pathogen interaction”. The identical DEGs were also significantly enriched more in “plant hormone signal transduction”, followed by “phenylpropanoid biosynthesis”.

Eight signaling transduction pathways related to hormones, auxin, cytokinin, gibberellin, abscisic acid (ABA), ethylene, brassinosteroid, jasmonic acid and salicylic acid (SA) (Additional files 9, 10, 11, Figs. S4-S6), were activated under salt stress, including 80 DEGs with 27 up-regulated genes and 53 down-regulated genes at 6 h, 80 DEGs with 36 up-regulated genes and 44 down-regulated genes at 24 h, and 55 DEGs with 13 up-regulated genes and 42 down-regulated genes DEGs at 72 h. In addition, 35 common DEGs with nine up-regulated genes and 26 down-regulated genes were significantly enriched in “plant hormone signal transduction” at 6 h, 24 h and 72 h, respectively. In the plant hormone signal transduction pathway, genes annotated as ethylene-responsive transcription factor (ERF), pathogenesis-related (PR) protein and auxin-related protein were identified (Additional file 3 : Table S3).

### Genes involved in antioxidant enzymes system and ion transport

A total of 17, 10 and 114 genes were annotated to SOD, CAT and POD, respectively. Most of these genes were mapped to the GO terms “removal of superoxide radicals”, “response to oxidative stress”, “cellular oxidant detoxification”, “oxidation-reduction process”, and others. In addition, we also identified genes related to ascorbate peroxidase and glutathione peroxidase involved in these terms (Additional file 4 : Table S4). Moreover, some DEGs were enriched in “oxidation-reduction process”. Besides, numerous genes related to sodium and potassium ion transport were also identified in *C. inerme* roots under NaCl treatment. Those genes were mapped to the GO terms “transmembrane transport”, “ion transport”, “integral component of membrane” and “potassium ion transport” (Additional file 4 : Table S4).

### Quantitative reverse transcription-polymerase chain reaction analysis of gene expression

**Fig. 10:**
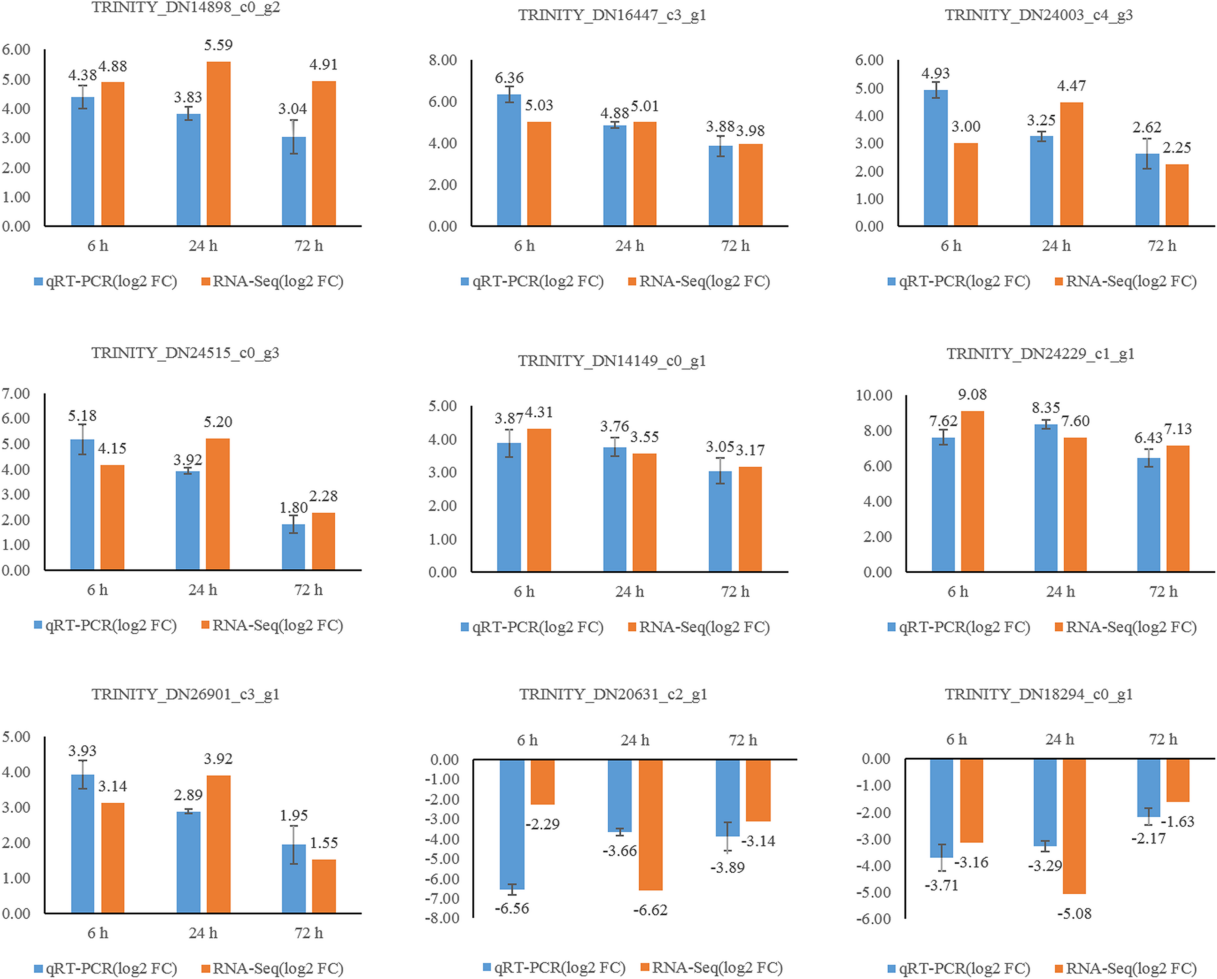
Analysis of the fold changes of nine candidate genes in *Clerodendrum inerme* determined by RNA-seq and qRT-PCR. The x-axis represents the time point after each treatment while the y-axis represents the log2 fold change. Graphs were generated by Microsoft Excel 2013

To further validate the results from the Illumina sequencing data, nine candidate DEGs were selected for quantitative reverse transcription-polymerase chain reaction (qRT-PCR) analysis of root samples that were treated with 400 mM NaCl for 0 h, 6 h, 24 h, and 72 h. In the four treatment stages, the trend of expression of the unigenes from qRT-PCR and RNA sequencing analysis were largely consistent (Fig. 10). These results demonstrate that the transcriptome data accurately reflects the response of *C. inerme* roots to salt stress.

## Discussion

Most studies have investigated long-term salt stress response in plants, and focused on various organs’ responses to salt stress, including leaves, roots and callus. For example, the dynamic transcriptome of poplar (*Populus simonii* × *Populus nigra*) leaves was investigated after exposure to NaCl for 3 d, 6 d and 9 d [44]. Transcriptomic changes to barley microspore-derived embryogenic callus was profiled in the period of cellular adaptation to stress after 21 d of culture in NaCl-supplemented medium [45]. Furthermore, the shoots and roots of salt mash euhalophyte, *Salicornia europaea*, collected from short- and long-term stress (exposure to 200 mM NaCl for 0 h, 12 h and 7 d) were used to perform high-throughput deep sequencing [46]. Although these analyses involved different treatments and organs, the genes and pathways of these species involved in salt tolerance at different stages were consistently revealed. Compared with aboveground organs, roots do not contain specialized structures such as salt glands, or photosynthetic systems. Given their simple structure, roots are highly sensitive to salt and are the primary site of perception to the soil environment. Roots are thus an essential organ to investigate a plant’s response to salt stress [47, 48]. A series of responses in roots are activated by short-term salt stress [49, 50]. These responses might be an effective means for a plant to resist salt stress. For these reasons, we performed a transcriptome analysis of *C. inerme* roots in response to short-term salt stress.

Physiological changes in roots at early stages after exposure to salt stress are also an indicator of substantial changes in salt-responsive gene expression in response to short-term salt stress. In *Populus tomentosa*, the accumulation of H_2_O_2_ and concentration of hormones changed significantly at 6 h, 12 h, and 24 h, and root samples at those time points were subjected to RNA-seq analysis to understand the short-term salt stress response, revealing the high enrichment of DEGs involved in H_2_O_2_ and hormone production [51]. In *Zoysia japonica*, oxidative stress caused by H_2_O_2_ was visible throughout roots after 30 min exposure to NaCl, and numerous candidate genes related to early responses to salt stress were revealed by de novo assembly of the Japanese lawngrass root transcriptome [52]. The activity of antioxidant enzymes in plants is closely related with tolerance to salt [53, 54]. Salt stress induces the accumulation of toxic compounds in cells, such as the superoxide radical and H_2_O_2_, both of which are reactive oxygen species (ROS). SOD converts the superoxide radical into H_2_O_2_ and H_2_O_2_ is further scavenged by POD, CAT or other enzymes [55–57]. The Na^+^/K^+^ balance in intracellular is also an important factor for plant salt tolerance. It is essential for the cytosol to maintain a low concentration of Na^+^ or a low Na^+^/K^+^ ratio in plant cells under salt stress [58]. In the present study, the activity of antioxidant enzymes in *C. inerme* roots increased rapidly after subjected to NaCl stress for 1, 6, 24, 48, 72, 120 h. SOD and CAT activity of *C. inerme* roots changed sharply or maintained the highest level after NaCl treatment at 6 h, 24 h, and 72 h. The dramatic changes of Na^+^ and K^+^ accumulation were also observed at 6 h, 24 h, and 72 h treatment. A series of salt response system might be activated at those time points. Using root samples at above time point, we identified genes related to antioxidant enzymes and ion transport, and those genes were involved in the ROS scavenger enzymatic system and ion transport in response to NaCl stress. This finding indicates that the time point at which antioxidant enzyme activity and ion accumulation changes significantly could serve as a valid marker to indicate substantial changes in salt-responsive gene expression in short-term adaptation to salt stress in *C. inerme* roots.

Significantly enriched GO terms and KEGG pathways may differ among plant species, but they still retain some similarities, despite the short- or long-term nature of salt stress. In *Raphanus sativus* roots treated with 200 mM NaCl for 48 h, DEGs were enriched in the GO terms “response to stimulus” and “single-organism process”, and the “plant-pathogen interaction” and “plant hormone signal transduction” pathways [59]. After treating *Helianthus tuberosus* roots with 150 mM NaCl for 15 d, most DEGs were mapped to “single-organism metabolic process” and “catalytic activity”. Numerous genes and proteins differentially expressed in *H. tuberosus* roots under salt stress had enriched GO terms “carbohydrate metabolism”, “ribosome activation” and “translation” [60]. In this study, the dominant enriched GO terms that were identified were “single-organism process”, “membrane” and “catalytic activity”, and more DEGs were significantly enriched in the “plant hormone signal transduction”, “carbon metabolism” and “plant-pathogen interaction” pathways. This result indicates that these GO terms and pathways were significantly involved in the plants response to salt stress, particularly in *C. inerme* roots.

Genes related to “plant hormone signal transduction” pathway has been verified to be involved in plant response to salt stress in several species*.* In *Iris halophila* shoots exposed to salt stress, the maximum number of DEGs displayed a significant enrichment in the “plant hormone signal transduction” pathway while the expression levels of genes related to cytokinin, gibberellin, ethylene, and SA signal transduction were also up-regulated in response to salt stress [61]. Compared with a salt-sensitive maize inbred line (L29), salt-tolerant inbred line L87 exhibited specific regulatory mechanisms related to salt tolerance. L87 exhibited increased expression of auxin, cytokinine, ethylene, ABA and SA biosynthetic genes involved in growth and protection. In contrast, L29 exhibited relatively lower expression of these genes [62]. Thus, genes, especially DEGs involved in the plant hormone signal transduction pathway, may also be an important category in research related to plant resistance to salt stress. We found that more DEGs were enriched in the “plant hormone signal transduction” pathway in the response of *C. inerme* roots to salt stress*.* Among them, nine DEGs performed up-regulated pattern at all the time points (6 h, 24 h and 72 h), so were 26 down-regulated genes. Those genes may be involved in a complex regulation system in the resistance to salt stress in *C. inerme* roots.

In *C. inerme* roots, we identified several DEGs annotated as ERF genes in the “plant hormone signal transduction” pathway after NaCl treatment. ERFs, which are plant-specific transcription factors, are involved in multiple biological processes, especially in abiotic stress [63]. *TaERF3*, a wheat ERF transcription factor, positively regulated the adaptation of wheat to salt and drought stress. Compared to untransformed wheat, the seedlings of *TaERF3*-overexpressing transgenic lines exhibited significantly enhanced tolerance to both salt and drought stress. Conversely, *TaERF3*-silenced wheat plants displayed more sensitivity to salt and drought stress than control plants [64]. Over-expression of the poplar transcription factor *ERF76* gene in tobacco enhanced its salt tolerance. Compared to the control, the transgenic line showed a significant increase in seed germination rate, plant height, root length, and fresh weight, as well as in relative water content, activity of antioxidant enzymes, and proline content after salt stress [65]. The role of ERFs in the response of *C. inerme* roots to salt stress needs additional verification.

PR proteins are always induced by various types of pathogens such as viruses, bacteria, and fungi and also by the application of chemicals that mimic the effect of pathogen infection or induce similar stresses. Based on their primary structure, serological activity, and biological activity, PR proteins have been classified into 17 different families, ranging from PR-1 to PR-17, and some members of those families are involved in the plant’s response to salt stress [66–68]. *AhSIPR10*, a salinity-induced PR class 10 protein, was isolated from callus cell lines of peanut (*Arachis hypogaea*). The overexpression of *AhSIPR10* enhanced tolerance to salt, heavy metal and mannitol-induced drought stress in transgenic tobacco line [69]. Transgenic plants overexpressing *AhSIPR10* in banana showed better photosynthetic efficiency and less membrane damage in the presence of NaCl and mannitol than wild type plants [70]. This result indicates that PR proteins participate in a variety of stress responses. We also identified several PR proteins in *C. inerme* roots after NaCl treatment. Those PR proteins were annotated on the SA signal transduction pathway of phenylethylamine metabolism. The differential expression level of those genes at all stages may indicate a certain function of PR proteins in the resistance of *C. inerme* roots to salt stress.

Auxin plays an important role in the regulation of plant growth and development, and auxin-related genes are also involved in plant stress and defense responses [71]. Under desiccation, cold, and salt stress, 41 members of auxin-related gene families were differentially expressed in rice under at least one abiotic stress condition while three genes were differentially expressed under all three stress conditions [72]. In *Saccharum narenga*, 175 genes encoding auxin-related proteins were identified in the leaves in response to drought treatment. DEGs encoding auxin response factors and auxin-induced proteins were up-regulated in sugarcane leaves in response to water deficit, and genes encoding indole-3-acetic acid were down-regulated in leaves from drought-treated samples compared to the control [73]. A halophyte is able to complete its life cycle at a high salt concentration. This may be closely related to the regulation of auxin. In *C. inerme* roots, we identified DEGs annotated as “auxin-induced protein”, “auxin-responsive protein”, “auxin response factor” and “auxin transporter-like protein” in the plant hormone signal transduction pathway. Those genes also participated in the response of *C. inerme* roots to salt stress. Exploration of the regulation of auxin-related genes in plants’ response to salt stress will uncover the crosstalk between signal transduction pathways involving phytohormones and plant salt tolerance.

## Conclusions

In the present study, the transcriptome of *C. inerme* roots was analyzed using RNA-seq technology to explore the mechanism involved in salt tolerance. The enriched GO terms and KEGG pathways that are involved in the response of *C. inerme* roots to short-term salinity were revealed. Genes related to the plant hormone signal transduction pathway were significantly involved in the response of *C. inerme* roots to saline conditions. Our findings provide numerous salt tolerant genes that can be applied in further research to improve the salt tolerance of functional plants and will enhance research on salt-tolerant mechanisms of halophytes.

## Methods

### Plant material and experimental design

Adult *C. inerme* plants were collected from Wenchang (108° 21′ to 111° 03′ E, 19° 20′ to 20° 10′ N), Hainan Province and maintained at South China Botanical Garden (SCBG) in Guangzhou, China, in 2017. Samples were collected and identified by Professor Shuguang Jian. A voucher specimen was deposited in the SCBG herbarium (voucher number 784713). Stem segments from mother plants were collected as explants and were washed under running tap water for 60 min. Excised stems were surface sterilized with 75% (v/v) ethanol for 30 s, rinsed in sterile distilled water, and dipped in 0.1% (w/v) mercuric chloride solution for 8 min followed by five or six rinses with sterile distilled water. After that, they were placed on sterile filter paper and allowed to dry briefly in air. Stems were cut into 2.0 cm long segments, each carrying one or more nodes, and inoculated onto Murashige and Skoog (MS) [74] basal medium containing 2.21 μM 6-benzyladenine (BA, Solarbio, Beijing, China) and 0.55 μM α-naphthaleneacetic acid (NAA, Sigma-Aldrich, St. Louis, USA). Axillary shoots developed within 30 d. Individuals axillary shoots (~ 2 cm long) were inoculated onto MS medium with 1.0 μM indole-3-butyric acid (IBA, Sigma-Aldrich) to initiate roots [75]. Plantlets with more than six roots were transferred to culture jars containing 15 mL of liquid MS medium and allowed to adapt for a week, then transferred to culture jars containing 15 mL of liquid MS medium with 400 and 600 mM NaCl, respectively. Roots at 0 h, 1 h, 6 h, 24 h, 48 h, 72 h, and 120 h after 400 mM NaCl treatment were harvested for analysis.

All media contained 30 g/L sucrose and 0.6% (w/v) agar, and pH was adjusted to 5.8–6.0, then autoclaved at 121 °C for 20 min. Culture jars (11 cm high; 6.5 cm diameter; 280 mL) were placed in an air-conditioned culture room at 25 ± 2 °C with a 12-h photoperiod under 100 μM m^− 2^ s^− 1^ fluorescent light (Philips, Tianjing, China) and 50–70% relative humidity.

### Antioxidant enzyme activity and H_2_O_2_ assay

Roots at 0 h, 1 h, 6 h, 24 h, 48 h, 72 h, and 120 h after exposure to NaCl treatment were harvested as ~ 0.1 g fresh weight (FW) with three biological replicates to assess the activity of antioxidant enzymes, including SOD (U/g FW, EC 1.15.1.1), POD (U/g FW, EC 1.11.1.7), and CAT (nmol/min/g FW, EC 1.11.1.6), and to determine H_2_O_2_ content (μmol/g FW), according to the instructions of a commercial chemical assay kit (Comin Biotechnology Co. Ltd., Suzhou, China) [76]. After one-way analysis of variance, treatment means were assessed by Duncan’s multiple range test in SPSS Statistics version 17.0 and considered to be significantly different from controls at *P* < 0.05.

### Determination of Na^+^ and K^+^ content

Harvested roots were dried at 80 °C for 48 h. Then the roots were grind to powder, and internal ions were extracted with 1 M HCl for 12 h. After the solution was filtered and volume to 50 mL, K^+^ and Na^+^ contents were measured with an atomic absorption spectrophotometer by flame mode [77].

### RNA extraction and cDNA library construction

Total RNA extraction was performed from three biological replicates at 0 h, 6 h, 24 h, and 72 h of NaCl-treated *C. inerme* roots using the Column Plant RNA_OUT_ Extraction kit (TIANDZ, Beijing, China) and treated with DNase I to remove genomic DNA, as suggested by the manufacturer. The concentration and quality of each sample were determined by agarose electrophoresis or an Agilent 2100 bioanalyzer (Agilent Technologies, Palo Alto, CA, USA). mRNAs were purified from total RNA using magnetic beads with oligo (dT) and fragmented into small pieces (200–300 bp). Then, the cleaved RNA fragments were primed with random hexamer primer for first-strand cDNA synthesis and second-strand cDNA synthesis. After purification, end repair, and ligation to sequencing adapters, 12 cDNA libraries of three biological replicates for each treatment were construct using the Illumina HiSeq 2500 platform by Personal Biotechnology Co., Ltd. (Shanghai, China).

### Sequencing and functional annotation

The percentage of unknown nucleotides as well as percentage of base recognition accuracy of more than 99.0% of bases (Q20) were calculated from raw reads. After cleaning the raw reads and discarding low quality reads, Trinity software (version 2.5.1, https://github.com/trinityrnaseq/trinityrnaseq/wiki; accessed March 15, 2018) was used for de novo assembly of high-quality reads. To annotate sequences obtained by de novo assembly, assembled transcripts were aligned to NCBI non-redundant protein sequences (NR, http://ftp.ncbi.nlm.nih.gov/blast/db/FASTA/; accessed March 15, 2018), Gene Ontology (GO, (https://www.blast2go.com/; accessed March 15, 2018), Kyoto Encyclopedia of Genes and Genome (KEGG, KAAS (http://www.genome.jp/tools/kaas/; accessed March 15, 2018), evolutionary genealogy of genes: Non-supervised Orthologous Groups (eggNOG, http://eggnog.embl.de/version_3.0; accessed March 15, 2018) and Swiss-Prot (http://www.uniprot.org/help/uniprotkb; accessed March 15, 2018) using BLASTX with a significance threshold of *E* ≤ 10^− 5^.

### Analysis of gene expression levels

Gene expression levels were quantified by RNA-seq by Expectation Maximization (RSEM) software (http://deweylab.github.io/RSEM/; accessed March 15, 2018). Gene expression level was calculated with the fragments per kilobase per transcript per million mapped reads method (FPKM). The number of transcripts after filtering was analyzed by density distribution and a boxplot graph to investigate the expression patterns among all samples. Pearson’s correlation between samples was calculated based on FPKM results to determine the operational stability and reliability of the experiment.

### Identification and functional annotation of DEGs

DEseq software (http://www.bioconductor.org/packages/release/bioc/html/DESeq.html; accessed March 15, 2018) was used to determine significant DEGs defined as a fold change (FC) ≥2 and *P* value < 0.05 at 6 h / 0 h, 24 h / 0 h, and 72 h / 0 h. Hierarchical clustering was performed to determine the expression pattern of DEGs in the 0 h, 6 h, 24 h, and 72 h libraries using Pheatmap software (https://cran.r-project.org/web/packages/pheatmap/index.html; accessed March 15, 2018). DEGs were mapped to each term of the GO database to detect significantly enriched GO terms with a corrected *P* value < 0.05 by the hypergeometric test method [78]. The number of DEGs at different levels was also calculated by the hypergeometric test to identify the main pathways involved in salt tolerance and to determine significantly enriched KEGG pathways at *P* < 0.05 [79].

### qRT-PCR analysis

Nine candidate DEGs, including seven up-regulated genes and two down-regulated genes in the enrichment KEGG pathway, were randomly chosen for qRT-PCR analysis to validate the transcriptomic data. qRT-PCR was performed with the ABI 7500 Real-time system (ABI, Alameda, CA, USA) using iTaq™ Universal SYBR Green Supermix (Bio-Rad, Foster, CA, USA). Actin of *C. inerme* was quantified as an internal control and the 2^−ΔΔCt^ method [80] was used to analyze differential expression. Gene-specific primers are listed in Additional file 5 : Table S5. Three biological replicates were performed for each candidate gene.

## Supplementary information

**Additional files 1: Table S1.** The significant enrichment GO terms at 6 h, 24 h, and 72 h. (XLSX 21 kb)

**Additional files 2: Table S2.** The significant enrichment KEGG pathway at 6 h, 24 h, and 72 h. (XLSX 16 kb)

**Additional files 3: Table S3.** The annotation DEGs on plant hormone signal transduction pathway. (XLSX 15 kb)

**Additional files 4: Table S4.** Genes related to antioxidant enzymes and ion transport. (XLSX 32 kb)

**Additional files 5: Table S5.** qRT-PCR primers. (XLSX 9 kb)

**Additional files 6: Fig. S1.** Density analysis of FPKM. (TIF 429 kb)

**Additional files 7: Fig. S2.** FPKM boxplot. (TIF 240 kb)

**Additional files 8: Fig. S3.** Correlation analysis of sample. (TIF 275 kb)

**Additional files 9: Fig. S4.** Plant hormone signal transduction pathway of DEGs at 6 h vs 0 h. (TIF 496 kb)

**Additional files 10: Fig. S5.** Plant hormone signal transduction pathway of DEGs at 24 h vs 0 h. (TIF 134 kb)

**Additional files 11: Fig. S6.** Plant hormone signal transduction pathway of DEGs at 72 h vs 0 h. (TIF 132 kb)

## Data Availability

All data generated or analyzed during this study are included in this published article and its supplementary information files. The RNA-seq data has been deposited in the Sequence Read Archives Database (https://www.ncbi.nlm.nih.gov/sra/; accessed November 7, 2018) under accession number PRJNA504939 (https://www.ncbi.nlm.nih.gov/sra/PRJNA504939).

## Abbreviations

ABA: Abscisic acid
BA: 6-Benzyladenine
CAT: Catalase
cDNA: Complementary deoxyribonucleic acid
DEG: Differentially expressed gene
DW: Dry weight
eggNOG: Evolutionary genealogy of genes: Non-supervised Orthologous Groups
ERF: Ethylene-responsive transcription factor
FC: Fold change
FPKM: Fragments per kilobases per million mapped reads
FW: Fresh weight
GO: Gene ontology
IBA: Indole-3-butyric acid
KEGG: Kyoto Encyclopedia of Genes and Genomes
MS: Murashige and Skoog
NAA: α-Naphthaleneacetic acid
NCBI: National Center for Biotechnology Information
NR: NCBI non-redundant protein sequences
PCR: Polymerase chain reaction
POD: Peroxidase
qPCR: Quantitative PCR
RNA: Ribonucleic acid
RNA-seq: High-throughput RNA-sequencing
ROS: Reactive oxygen species
RSEM: RNA-seq by Expectation Maximization
SA: Salicylic acid
SOD: Superoxide dismutase
