# Supplementary figures and images for: RNA-Seq analysis of Clerodendrum inerme (L.) roots in response to salt stress
Source: BMC Genomics. 2019 Oct 10;20:724. doi: 10.1186/s12864-019-6098-y (PMC6785863; doi:10.1186/s12864-019-6098-y)

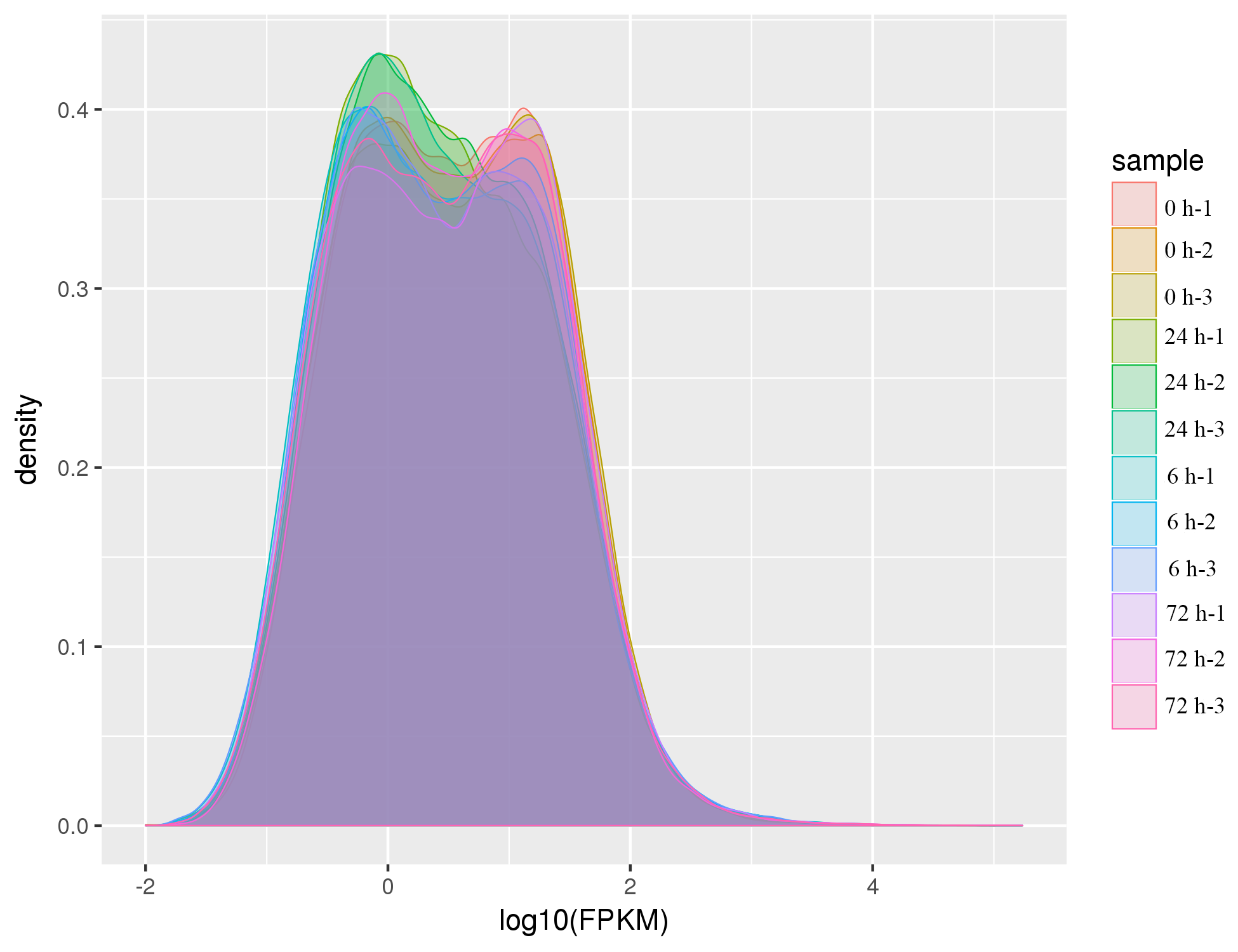

Supplement: Supplementary file 6 — Additional files 6: Fig. S1. Density analysis of FPKM. (TIF 429 kb) [file 12864_2019_6098_MOESM6_ESM.tif]

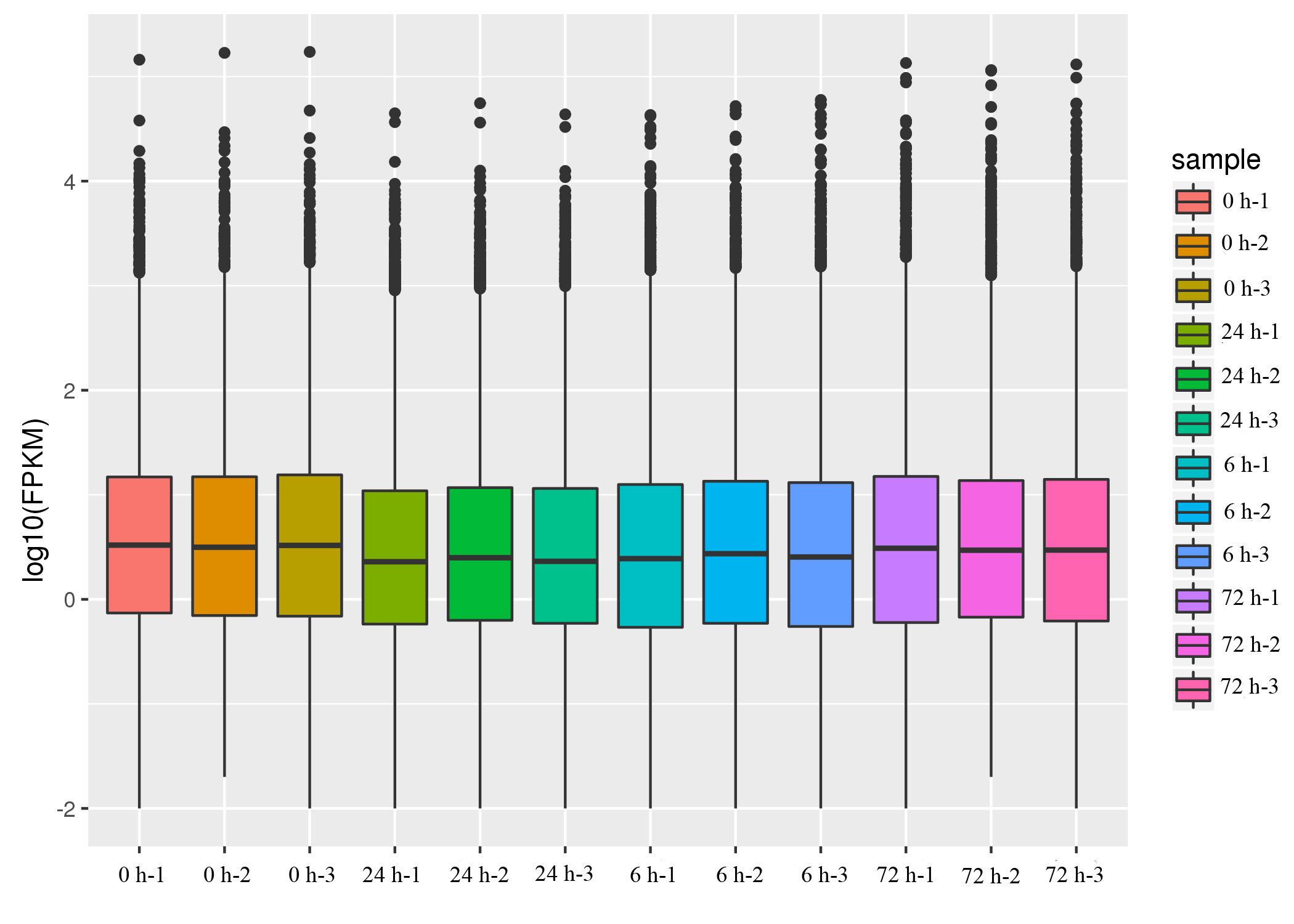

Supplement: Supplementary file 7 — Additional files 7: Fig. S2. FPKM boxplot. (TIF 240 kb) [file 12864_2019_6098_MOESM7_ESM.tif]

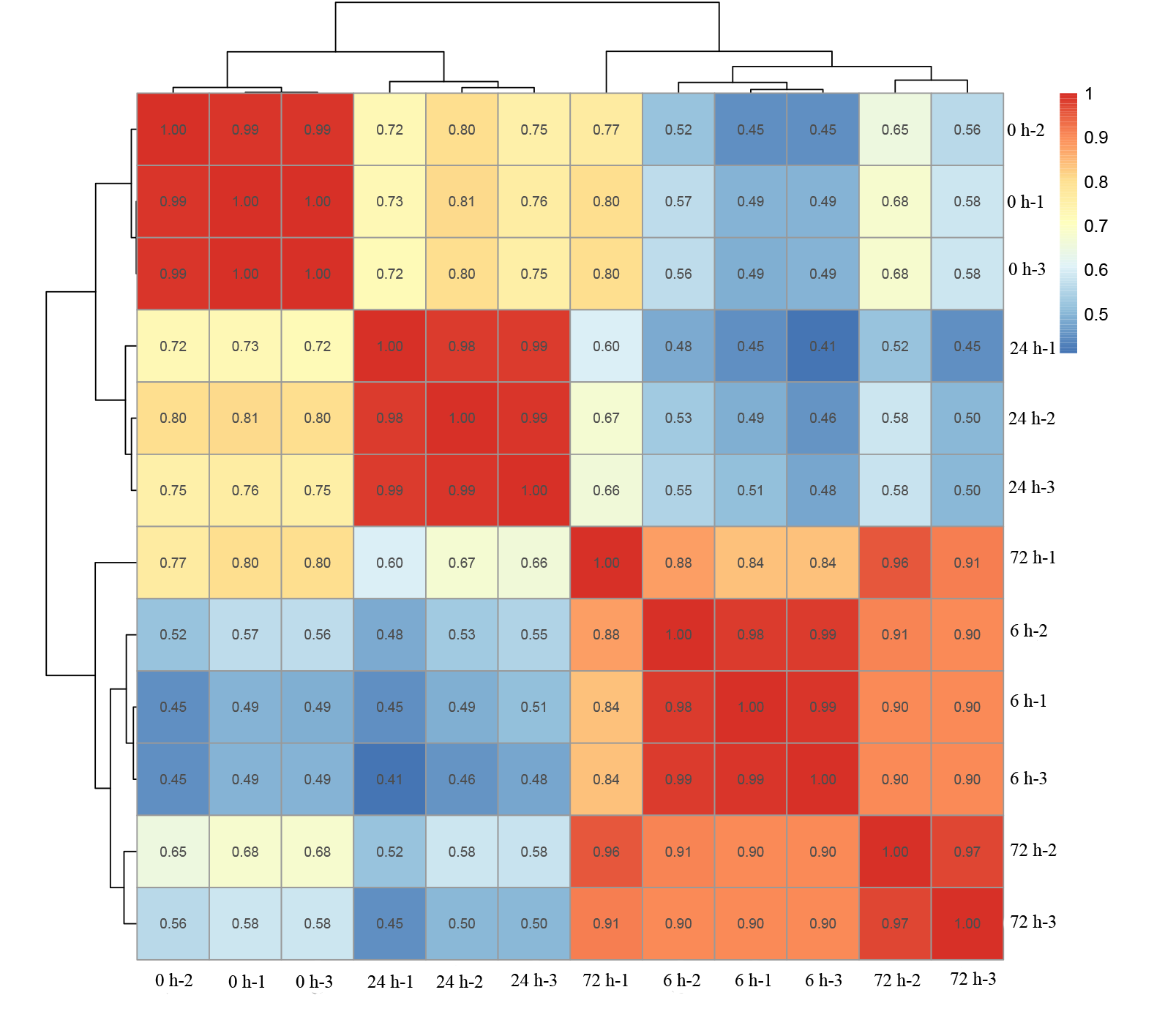

Supplement: Supplementary file 8 — Additional files 8: Fig. S3. Correlation analysis of sample. (TIF 275 kb) [file 12864_2019_6098_MOESM8_ESM.tif]

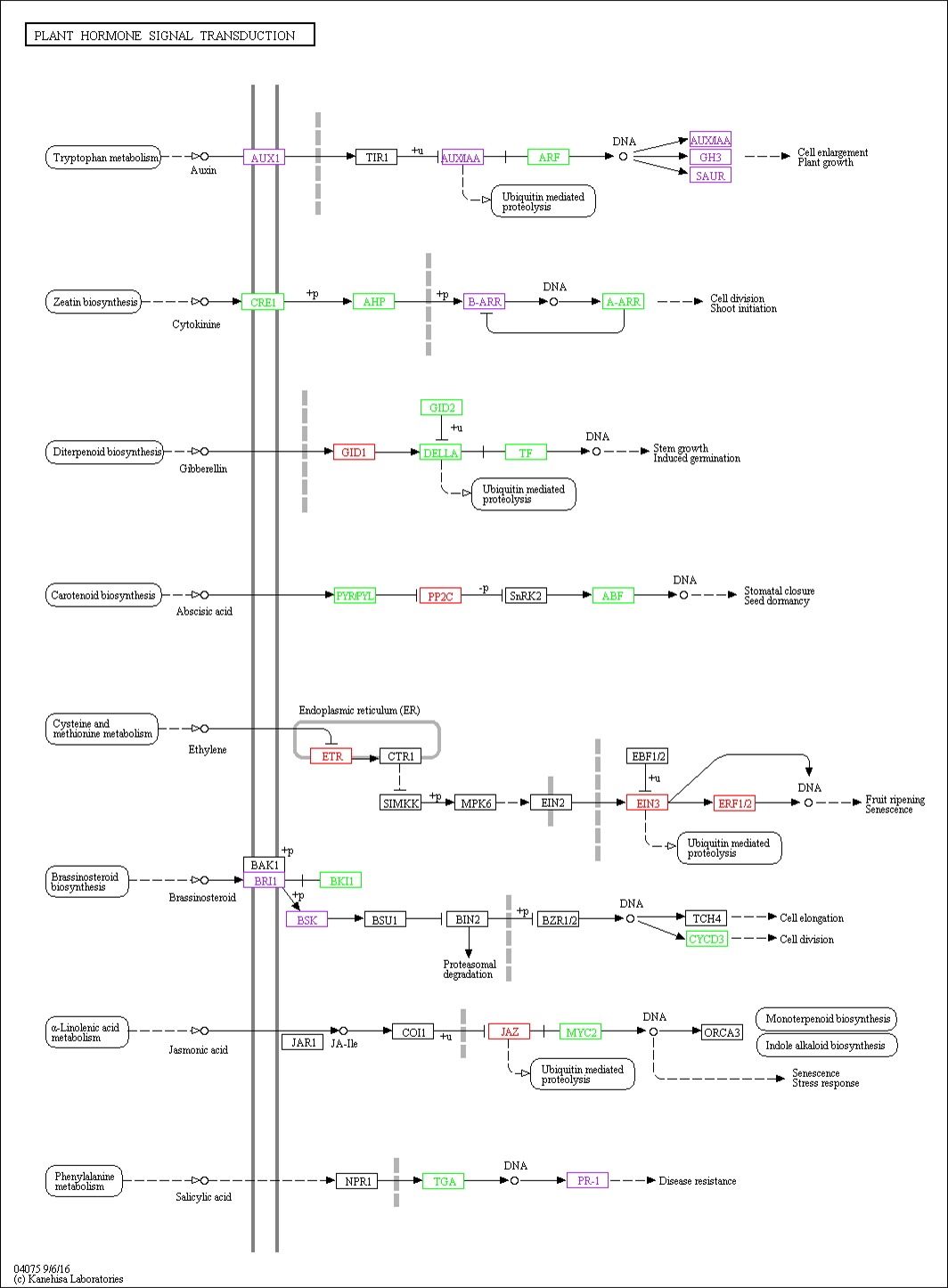

Supplement: Supplementary file 9 — Additional files 9: Fig. S4. Plant hormone signal transduction pathway of DEGs at 6 h vs 0 h. (TIF 496 kb) [file 12864_2019_6098_MOESM9_ESM.tif]

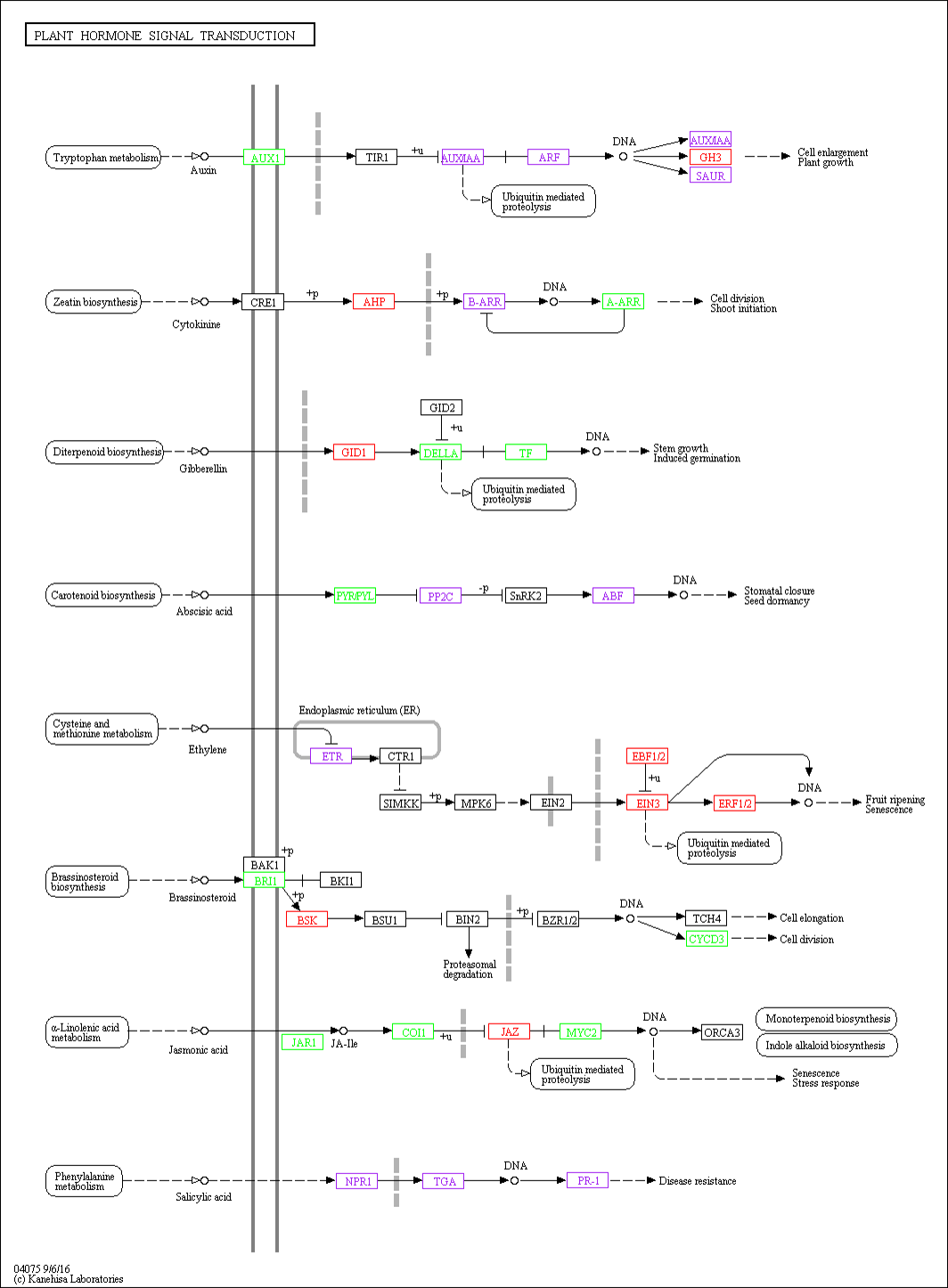

Supplement: Supplementary file 10 — Additional files 10: Fig. S5. Plant hormone signal transduction pathway of DEGs at 24 h vs 0 h. (TIF 134 kb) [file 12864_2019_6098_MOESM10_ESM.tif]

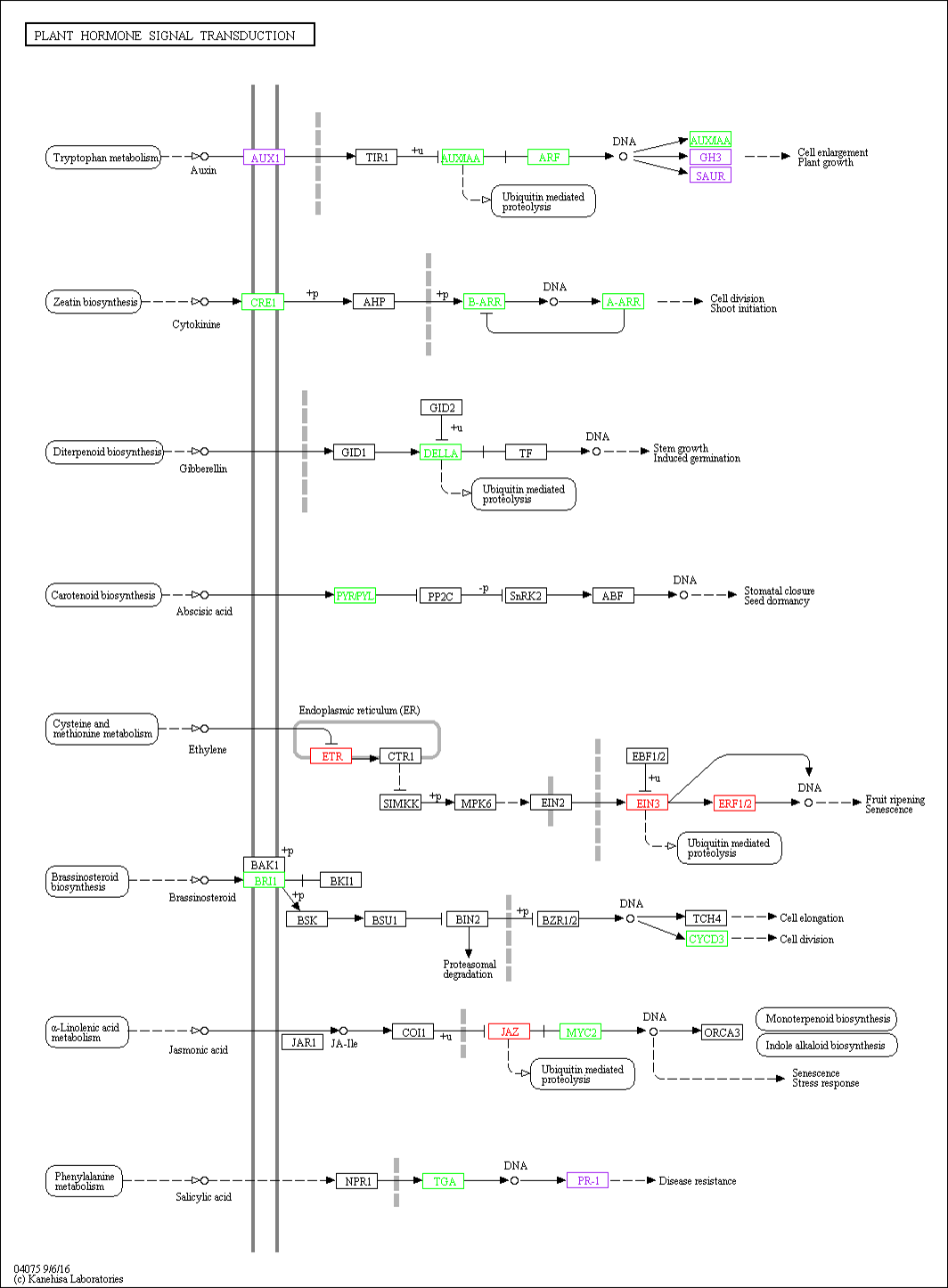

Supplement: Supplementary file 11 — Additional files 11: Fig. S6. Plant hormone signal transduction pathway of DEGs at 72 h vs 0 h. (TIF 132 kb) [file 12864_2019_6098_MOESM11_ESM.tif]
